# HMG-CoA reductase from Camphor Tulsi (*Ocimum kilimandscharicum*) regulated MVA dependent biosynthesis of diverse terpenoids in homologous and heterologous plant systems

**DOI:** 10.1038/s41598-017-17153-z

**Published:** 2018-02-23

**Authors:** Shilpi Bansal, Lokesh Kumar Narnoliya, Bhawana Mishra, Muktesh Chandra, Ritesh Kumar Yadav, Neelam Singh Sangwan

**Affiliations:** 1 Department of Metabolic and Structural Biology, CSIR-Central Institute of Medicinal and Aromatic Plants, Lucknow, 226015 UP India; 2 Academy of Scientific and Innovative Research (AcSIR), CSIR-Human Resource Development Centre Campus, Sector- 19, Kamla Nehru Nagar, Ghaziabad, Uttar Pradesh 201002, India

## Abstract

*Ocimum kilimandscharicum* is unique in possessing terpenoids whereas other *Ocimum* species are renowned for phenylpropanoids as major constituents of essential oil. The key enzyme of MVA/terpenoid metabolic pathway *viz* 3-hydroxy-3-methylglutaryl Co-A reductase (*OkHMGR*) of 1.7-Kb ORF encoding ~60-kDa protein was cloned from *O*. *kilimandscharicum* and its kinetic characteristics revealed the availability of HMG-CoA as a control point of MVA-pathway. Transcript profiling of the *OkHMGR* elucidated tissue-specific functions of the gene in flower and leaf tissues in accumulation of terpenoidal essential oil. *OkHMGR* was differentially regulated in response to exposure to methyl-jasmonate, salicylic-acid, and stress conditions such-as salt and temperature stress, demonstrating its key role in managing signaling and stress-responses. To elucidate its functional role, *OkHMGR* was transiently over-expressed in homologous and heterologous plants such as *O*. *sanctum*, *O*. *basilicum*, *O*. *gratissimum*, *Withania somnifera* and *Artemisia annua*. The over-expression and inhibition dual strategy revealed that the additional *OkHMGR in-planta* could afford endogenous flow of isoprenoid units towards synthesis of terpenoids. The present study provides in-depth insight of *OkHMGR* in regulation of biosynthesis of non-plastidal isoprenoids. This is first report on any gene of MVA/isoprenoid pathway from under-explored Camphor Tulsi belonging to genus Ocimum. Studies also suggested that *OkHMGR* could be a potential tool for attempting metabolic engineering for enhancing medicinally important terpenoidal metabolites in plants.

## Introduction

**Figure 1:**
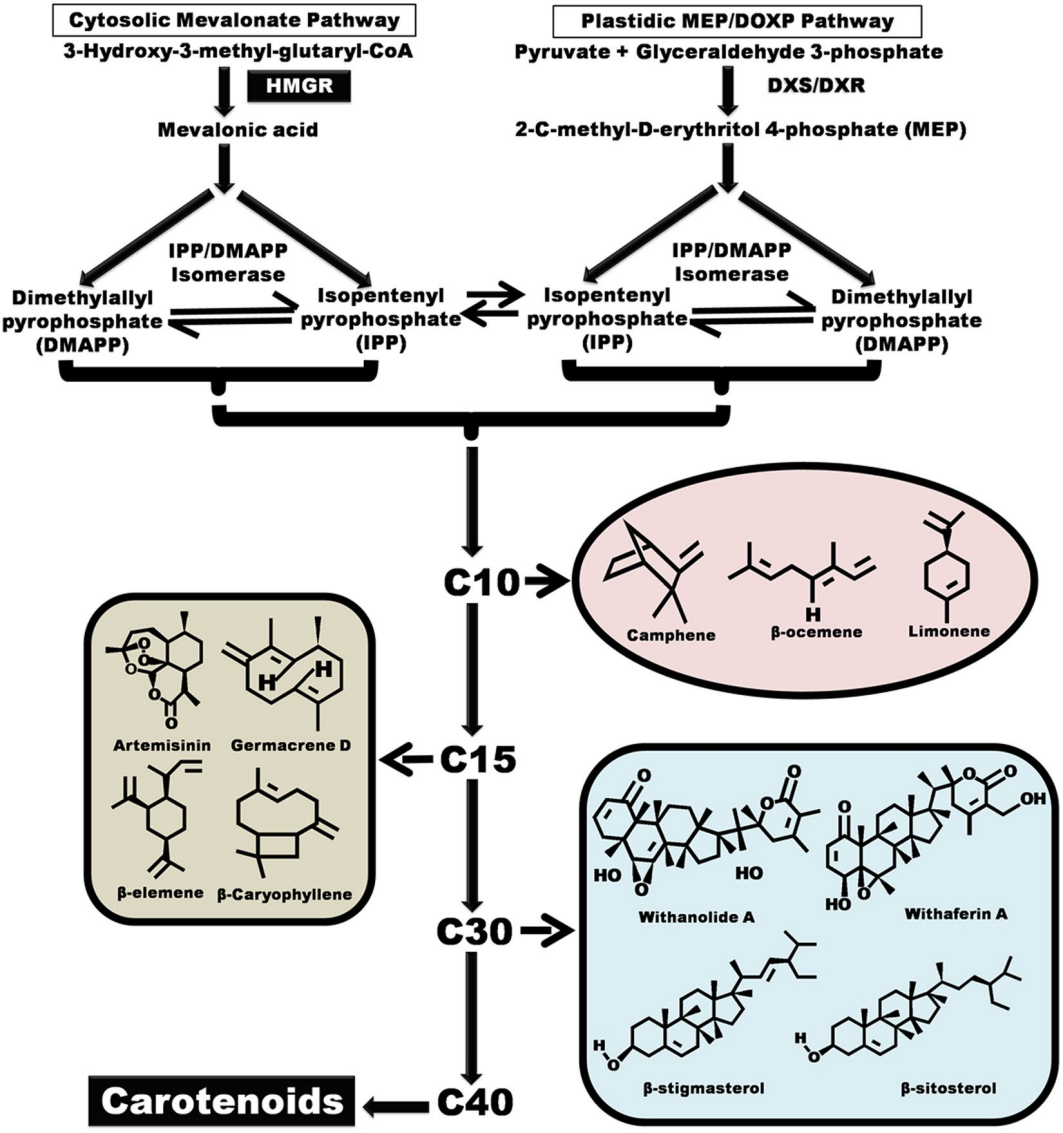
Schematic diagram of terpenoid biosynthetic pathway through mevalonate (MVA) as well as methyl-erythritol-phosphate (MEP) pathway.

*Ocimum* (Tulsi), an important member of Lamiaceae family comprises of about 150 species that are the inhabitants of tropical and warm temperate regions of Asia, Central and South Africa^1^. The Tulsi has been used as a source of medicinal preparations since ages, mentioned in various ancient literatures such as Charak Samhita, Rigveda etc. In Sanskrit the meaning of Tulsi is “one that is incomparable”. It is classified as a “Rasayana,” the herb which improves the health. The prominently used species of genus Ocimum are *O*. *basilicum*, *O*. *sanctum*, whereas *O*. *gratissimum* and *O*. *kilimandscharicum* are less explored^2^. *O*. *kilimandscharicum* is known for camphor as major constituent in its essential oil and is a perennial herb, native of East Africa and is also found in India, Turkey and Thailand^3,4^. The plant is rich in aromatic essential oil that makes it economically and medically important. *O*. *kilimandscharicum* contains pool of various secondary metabolites with a major proportion of monoterpenoids (≥ 90.0%) out of which oxygenated monoterpenoids may constitute upto 70% while hydrocarbons may constitute about upto 25%^5^. It has been reported that the essential oil content in Camphor Tulsi varied between 0.70–2.0% on dry weight basis^6^. Seed and leaf essential oil of *O*. *kilimandscharicum* also contains minor proportions of other monoterpenoids such as (α-pinene, camphene, β-myrcene along with sesquiterpenoids s such as trans-caryophyllene, germarcrene‐D^4^. Presence of a wide range of these secondary metabolites makes *O*. *kilimandscharicum* a valuable material to be used as medicinal bio-resource^7^. Phytomolecules such as methyl chavicol, 1,8-cineole, eugenol, (E)-bisabolene, terpineol, linalool, (Z)-cinnamic acid methyl ester, camphor etc are reported to possess various antimicrobial, antispasmodic, bactericidal, carminative, anti-helminthic, hepatoprotective, antiviral and larvicidal activities^6–8^. Camphor is the most distinct compound of the plant which may serve various medicinal and industrial purposes^5–8^. The terpenoid metabolites are produced by the plant through MEP (2C-methyl-D-erythritol 4-phosphate) and MVA (mevalonic acid) pathway in plastids and cytosol, respectively^9^. Both the pathways are actively involved in the biosynthesis of isoprenoid moieties operative from their respective compartments^10,11^. Mono, di-, tetra- terpenoids are mainly biosynthesized by MEP pathway whereas MVA pathway is reported for the biosynthesis of sesquiterpenoids and triterpenoids^10,11^. Our earlier studies on *W*. *somnifera* showed that the mevalonate pathway contributes dominantly to the synthesis of IPP than non-mevalonate pathway and HMG-CoA to mevalonic acid *via* HMGR enzyme is the rate-determining step^12,13^. Undoubtedly, HMGR gene governs the MVA pathway derived isoprenoids and plays a key role in isoprenoid biosynthesis. HMGR gene has been isolated from number of plant species including *W*. *somnifera*, *S*. *miltiorrhiza*, *A. indica* and other plants^12–20^. Different isoforms of HMGR have also been reported in cotton and tomato, which might have different roles to play under the influence of different environmental and developmental conditions^15,16^. HMGR is responsible for regulating the production of diverse terpenoids virtually in all plants and holds central regulatory position in terpenoid bearing plants (Fig. 1). Considering the key dominance of HMGR in terpenoidal essential oil biosynthesis, in the present study, we report the cloning and characterization of HMGR from *O*. *kilimandscharicum*, functional expression in *E*. *coli* and plants along with its association with secondary metabolite biosynthesis. To the best of our knowledge, this is the first report of kinetic and *in planta* study of HMGR from *Ocimum* species. The study provides a novel *OkHMGR* which can be used as a powerful sequence to drive MVA led, isoprenoids synthesis in medicinally and aromatically important plants.

## Results

### Cloning of *OkHMGR*

A 500 bp amplicon was obtained from *O*. *kilimandscharicum* leaf tissues using degenerate primers (Supplementary Fig. 1A). This partial amplicon was used for designing RACE primers. PCR amplification with 5′ RACE and 3′ RACE primers gave amplicon of 700 bp and 1000 bp respectively (Supplementary Fig. 1B,C). Assembly of all these partial and RACE amplified products resulted in a full length *OkHMGR*. Once the full length sequence was obtained gene specific full length primers were designed by incorporating restriction enzymes *Sal*I and *Hind*III at 5′ and 3′ terminus. The ORF search confirmed 1698 bp coding region encoding a polypeptide of 565 amino acids. The theoretical pI of the protein is 7.10 and molecular weight is 60.53 kDa (Supplementary Table 1).

### Sequence retrieval and phylogenetic studies

**Figure 2:**
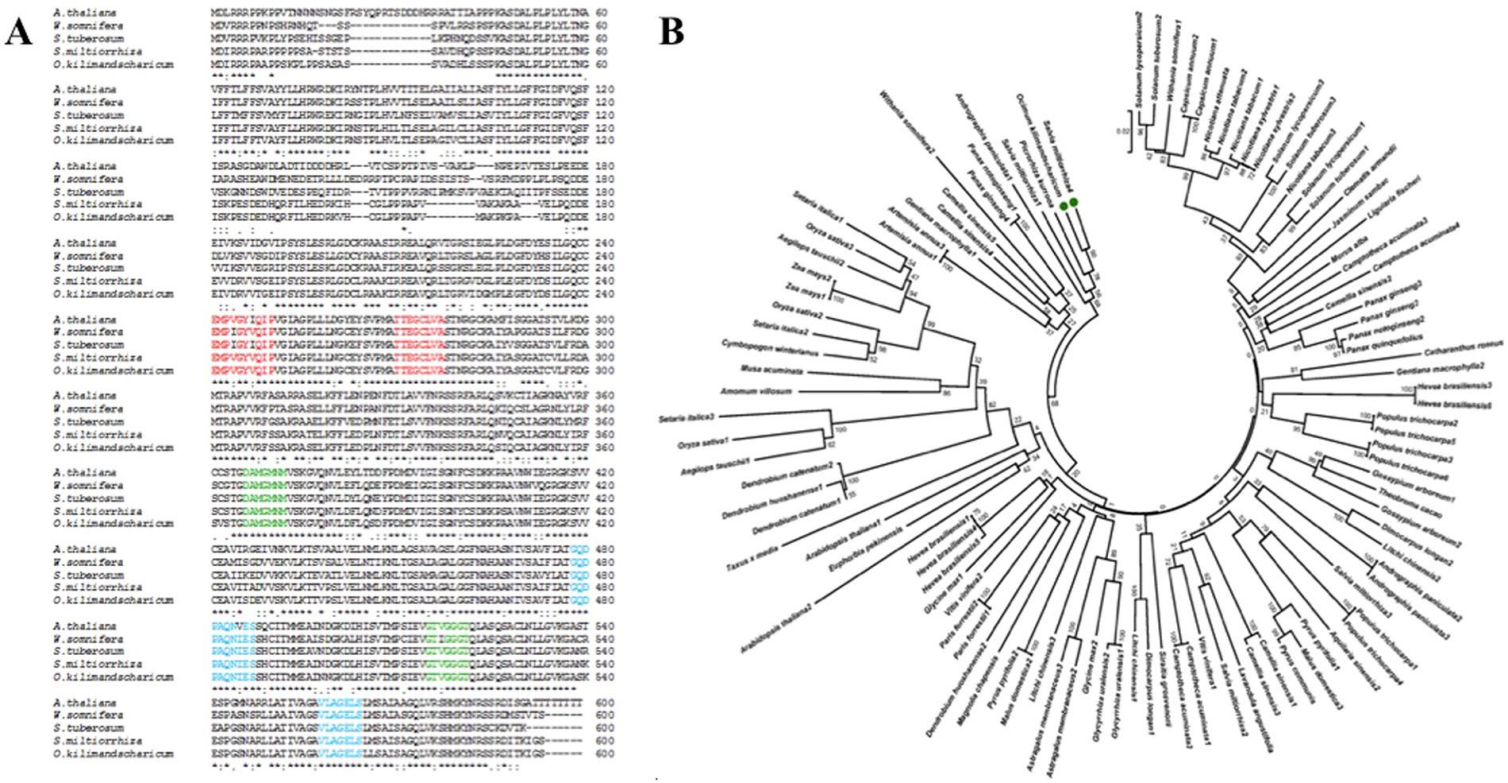
Multiple sequence alignment and phylogenetic analysis of OkHMGR. (**A**) Multiple sequence alignment of OkHMGR and visualization of different motifs. Three sets of motifs, each having two motifs were detected. NADP(H) binding motifs (green), HMG-CoA binding motifs (red), and plant specific motifs(blue). The consensus sequences of NADPH binding motif are [DAMGMNM] & [GTVGGGT], HMG-CoA binding motifs are [EMPVGYVQIP] & [TTEGCLVA] and plant specific motifs are [GQDPAQN] & [VLAGELS] (**B**) Phylogenetic analysis of 108 plant HMGRs from 55 different species analysed by neighbor joining approach, with 1000 bootstrap replicates.

Full length *OkHMGR* exhibited maximum similarity with HMGR of *Salvia* *miltiorrhiza* (94%) followed by *Andrographis paniculata* (86%), *Picrorhiza kurrooa* (86%), *Panax ginseng* (83%), *W. somnifera* (78%), *Arabidopsis thaliana* (78%) and *Nicotiana tabacum* (75%). Plants HMGR possess HMG-CoA binding motifs (EMPIGYVQIP) & (TTEGCLVA) and NADPH-binding motifs (DAMGMNM) & (GTVGGGT) which were also found in the OkHMGR (Fig. 2A). Likewise motifs (GQDPAQN) & (VLAGELS) were found to be highly conserved and appear to be representative motifs for plant HMGR as these were found to be missing in human HMGRs (Fig. 2A). Multiple sequence alignment analysis of 108 sequences of 55 different plant species coding for HMGR retrieved from database and their phylogeny along with the sequence from *O*. *kilimandscharicum* (OkHMGR) exhibits closeness to *S*. *miltiorrhiza* and the functional motifs were very similar to other plant HMGRs (Fig. 2B).

Secondary structure prediction study revealed presence of 20 alpha helices, 12 β-strands intervened by a number of coils (Supplementary Fig. 2). Target P1.1 server observed that OkHMGR is a cytosolic protein and theoretical pI determined was 7.10. The incidence of both aspartic acid and glutamic acid (negatively charged amino acids) and arginine and lysine (positively charged amino acid) were 53 each. The instability index and aliphatic index were 43.45 and 95.46 respectively (Supplementary Table 1). These features are key determinant of regulating catalytic activities of HMGR protein.

### Heterologous expression, purification and kinetic characterization of OkHMGR

**Figure 3:**
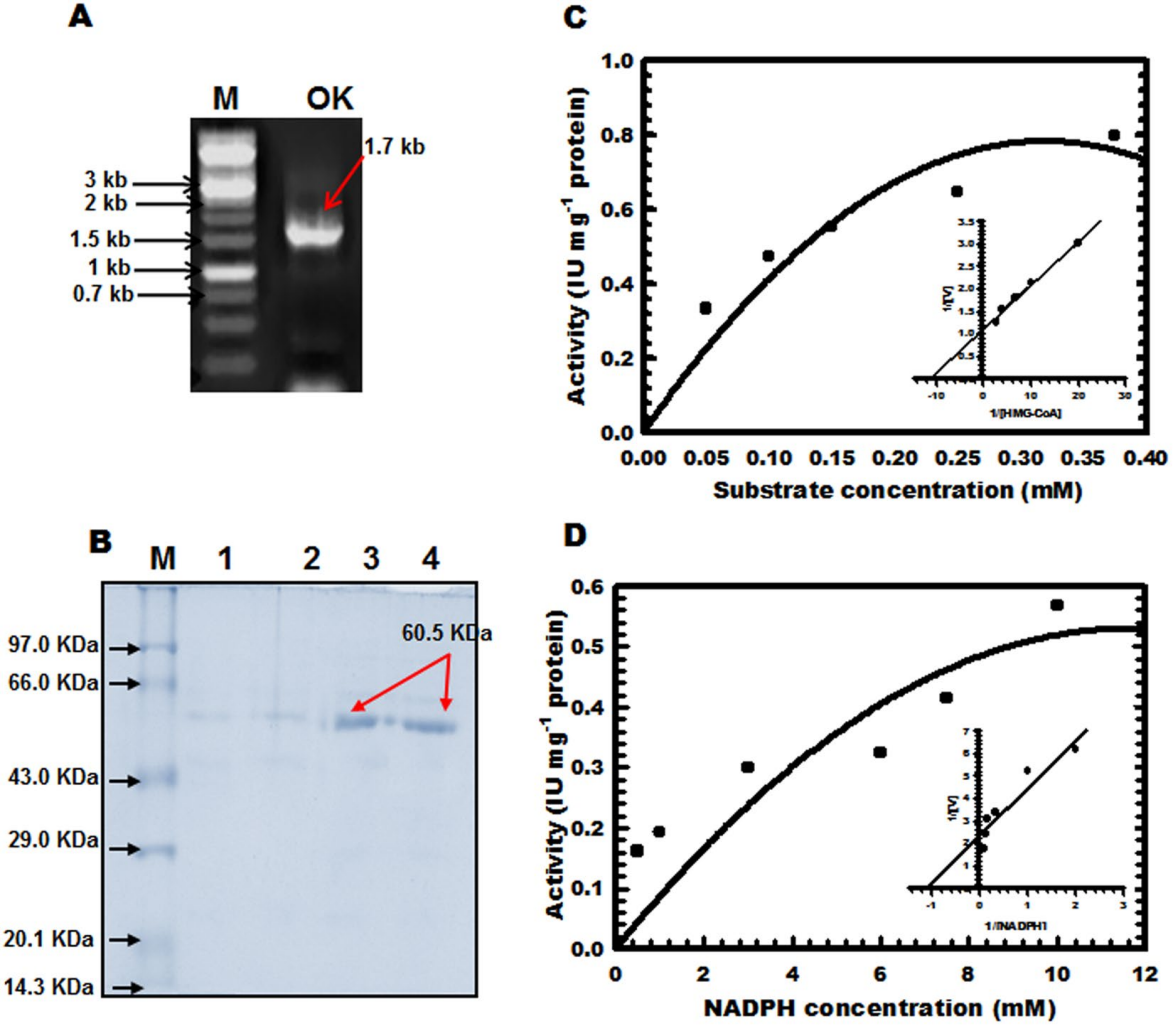
Molecular and biochemical analysis of *OkHMGR* gene and protein. (**A**) PCR amplification product (1.7Kb) amplified with gene specific primers. (M-1Kb ladder, OK-amplified product), (**B**) SDS-PAGE analysis of His-tagged recombinant OkHMGR (lane 1: protein molecular weight marker; lane 1–4 OkHMGR enzyme), (**C**) Substrate saturation curves for HMG-CoA and (**D**) Substrate saturation curves for NADPH.

Full length *OkHMGR* (approximately 1.7 kb; Fig. 3A) was cloned in pET28a expression vector. Recombinant OkHMGR (60 kDa) protein (Fig. 3B) was biochemically characterized for various catalytic and kinetic properties. OkHMGR activity was found maximum in potassium phosphate buffer followed by sodium phosphate and Tris buffer (Supplementary Fig. 3). OkHMGR activity increased with the increase in pH from 5.0 to 7.0 with maximum activity at about 7.0, thereafter a drastic reduction in activity was noticed (Supplementary Fig. 4). The OkHMGR enzyme showed a hyperbolic curve both for substrate and cofactor as revealed by substrate saturation studies. Various cations (Mn^2+^, Li^2+^, K^+^, Na^+^, Ca^2+^ and Mg^2+^) introduced in the enzyme assay influenced the catalytic activity of the enzyme. It was observed that addition of K^+^ increased the activity of enzyme (Supplementary Fig. 5). Using Lineweaver-Burk plot, Michaelis constant (K_m_) and the maximum reaction velocity (V_max_) were determined (Fig. 3C,D). The K_m_, V_max_, K_cat_ and K_cat_/K_m_ values for HMG-CoA were 0.088 mM, 0.909µmole min^−1^ mg^−1^ enzyme protein, 31.13 s^−1^ and 3.53 × 10^5^ M^−1^ s^−1^ respectively while for NADPH values were 0.903 mM (through LB plot), 0.425µmole min^−1^ mg^−1^ enzyme protein, 14.53 s^−1^ and 1.6 × 10^4^ M^−1^ s^−1^ respectively.

### Modulation of *OkHMGR*

**Figure 4:**
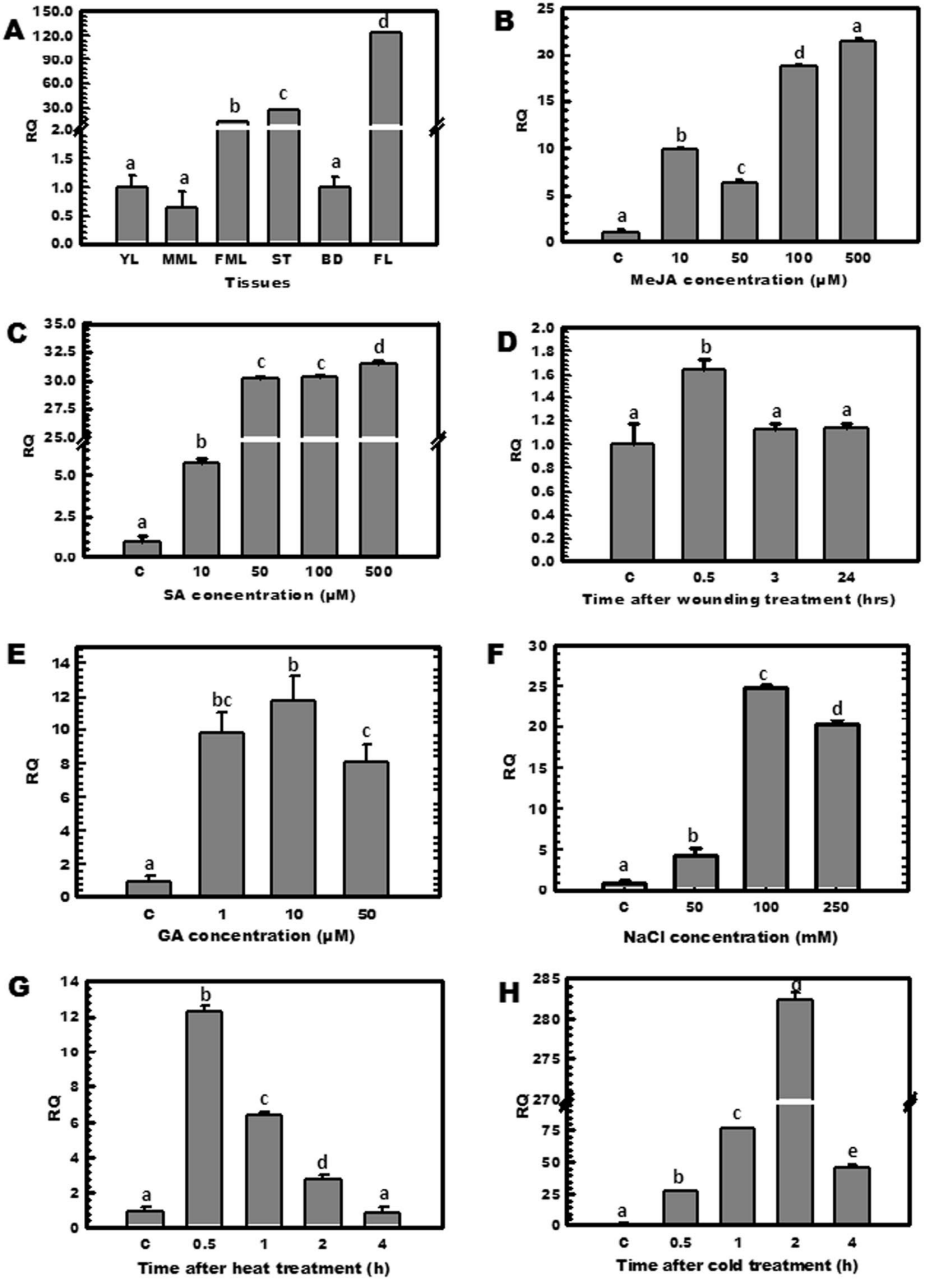
Expression profiles of *OkHMGR* in different tissues of plant as well as under the influence of elicitors and various stress conditions. Relative expression profiling of *OkHMGR* (**A**) in different tissues of *O*. *kilimandscharicum*,(YL-Young leaf, MML-Middle mature leaf, FML-Fully mature leaf, ST-Stem, BD-Bud, FL-Flower), (**B**) in response to different strength of methyl jasmonate (0, 10, 50, 100 and 500 µM), (**C**) in response to different strength of salicylic acid (0, 10, 50, 100 and 500 µM), (**D**) on wounding (0, 0.5, 3 and 24 hrs), (**E**) under the influence of gibberellic acid (0, 1, 10 and 50 μM), (**F**) after NaCl treatments (0, 50, 100 and 250 mM), (**G**) under heat stress for different time period (0, 0.5, 1, 2 and 4 hrs), (**H**) and after exposure to cold stress (0, 0.5, 1, 2 and 4 hrs).

The abundance of *OkHMGR* transcripts in *O*. *kilimandscharicum* was found to be highest in the flower followed by other tissues (Fig. 4A). *OkHMGR* was found to be regulated by methyl jasmonate (MeJA) and salicylic acid (SA) as exhibited by the exogenous supplementation studies. A substantial increase in transcript abundance was observed with MeJA (Fig. 4B), and with SA (Fig. 4C). Whereas, mechanical injury of plants, also modulated the *OkHMGR* expression by 1.6 fold (Fig. 4D).

Similarly, the presence of GA_3_ (gibberellic acid) hormone for 3 h, elevated the expression of *OkHMGR* transcripts (Fig. 4E). In addition, salt stress showed substantial increase in the expression of *OkHMGR*. After 3 h of treatment, transcript levels increased by 4 to 24 folds at 50 and 100 mM, with a decrease to 20 folds at 250 mM concentration (Fig. 4F). Interestingly, exposure to heat stress at 55 °C for different time intervals resulted in the substantial up-regulation of gene (Fig. 4G). Likewise, the cold stress treatment showed that the enzyme is under regulation by cold condition (Fig. 4H).

### Gene expression and secondary metabolite content in different *Ocimum* species

**Figure 5:**
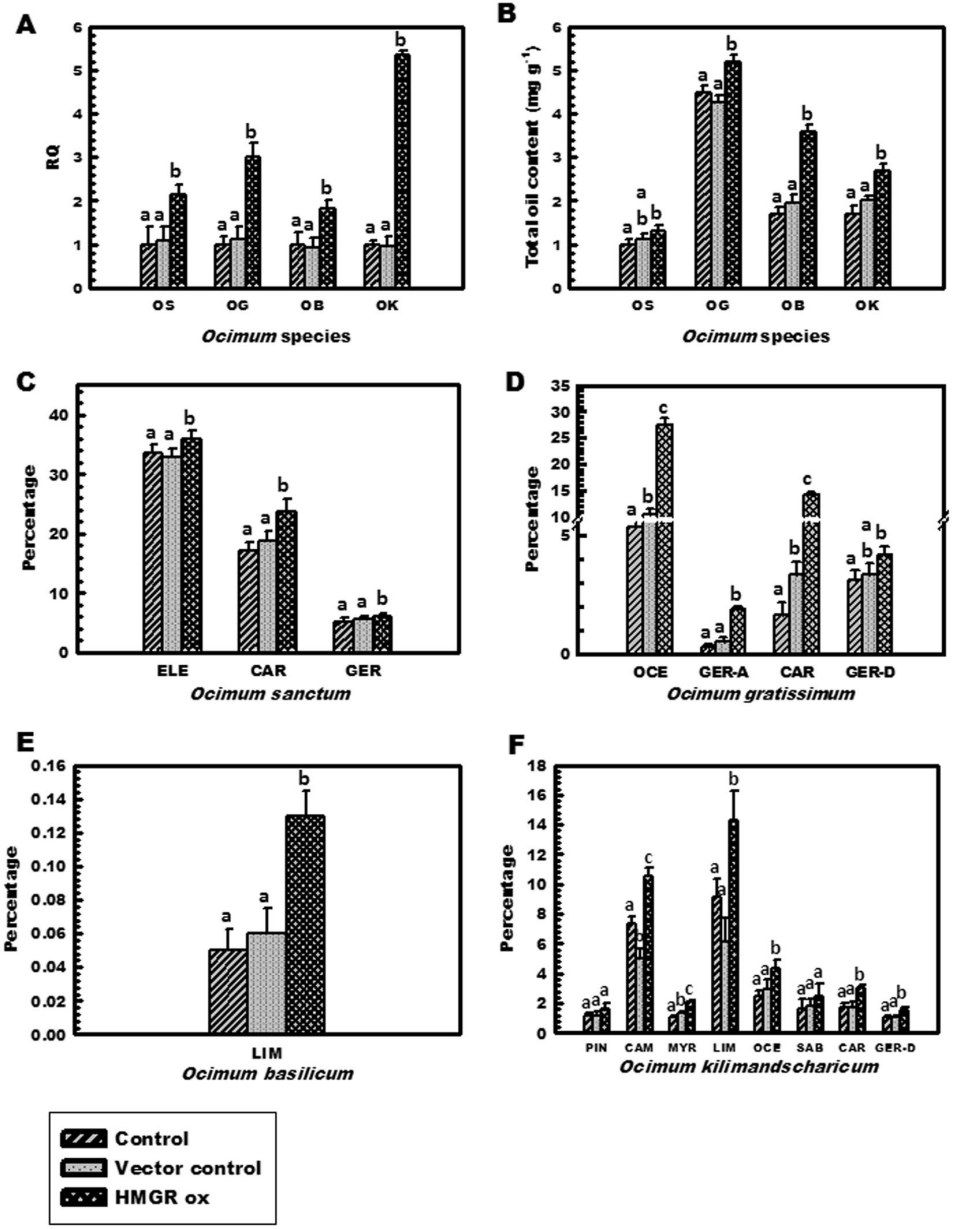
Relative expression profiles of *OkHMGR* transcript and secondary metabolite content in control, vector control and over-expressed lines of different species of *Ocimum* (OS: *O*. *sanctum*, OG: *O*. *gratissimum*, OB: *O*. *basilicum*, OK: *O*. *kilimandscharicum*). (**A**) *OkHMGR* mRNA transcript levels, (**B**) Total essential oil content (mgg^−1^) in above mentioned four species of *Ocimum*. Change in essential oil constituent’s percentage (%) in (**C**) *O*. *sanctum*, (**D**) *O*. *gratissimum*, (**E**) *O*. *basilicum* and (**F**) *O*. *kilimandscharicum*. ELE- β-elemene, CAR- caryophyllene, GER- germacrene D, OCE-β-ocimene, GER A- germacrene A, LIM- limonene, PIN- α-pinene, CAM- camphene, MYR- myrecrene, TER- gamma-terpinene, SAB- cis-sabinene hydrate. Control represents the untransformed tissues, vector control represents the tissues transformed with pCAMBIA1303 vector and HMGRox represents tissues transformed with *OkHMGR*.

We observed that the transient over-expression of *OkHMGR* in different species of *Ocimum* namely; *O*. *sanctum*, *O*. *basilicum* and *O*. *gratissimum* resulted in increased transcript abundance of *OkHMGR*, with about 2.1, 1.8 and 3 folds respectively, compared to their respective controls (Fig. 5A). The values of RQ were correlated well with total essential oil content, isolated from transformed and control plants (Fig. 5B). Increased essential oil content in transiently transformed tissues suggests that *HMGR* may have a direct role in essential oil biosynthesis. In *O*. *sanctum* remarkable increase in quantity of sesquiterpenoids such as beta-elemene (7.4%), caryophyllene (37.87%) and germacrene D (19.1%) were observed (Fig. 5C). Interestingly, another species *O*. *gratissimum* showed tremendous increase in both sesquiterpenoids and monoterpenoid constituents. The compounds with increased content were beta-ocimene, germacrene A, caryophyllene, and germacrene D (Fig. 5D), whereas in *O*. *basilicum* limonene was detected as major monoterpenoid constituent (Fig. 5E). In *O*. *kilimandscharicum*, transformation of the plant with *OkHMGR*, triggered the expression of *HMGR* and thereby the production of terpenoidal essential oil was also enhanced. Transcript abundance of *OkHMGR* in over-expressed lines displayed about 5.36 folds increment when compared with untransformed control (Fig. 5A,B). When the individual constituents of essential oil were analysed by GC, marked increase in sesquiterpenoids and some of the monoterpenoids was observed. Sesquiterpenoids which were found increased after transformation event were germacrene A (75%), β-caryophyllene (18.43%), germacrene D (9.71%) (Fig. 5F). The mRNA transcript levels and essential oil content on comparison with controls (untransformed and vector control) lines did not show significant deviation from the results obtained, i.e both the controls showed almost similar response (Fig. 5) thereby clearly demonstrating that increase in gene expression and secondary metabolite content was due to *OkHMGR* over-expression.

### Gene expression and secondary metabolite content in *Artemisia annua* and *Withania somnifera*

**Figure 6:**
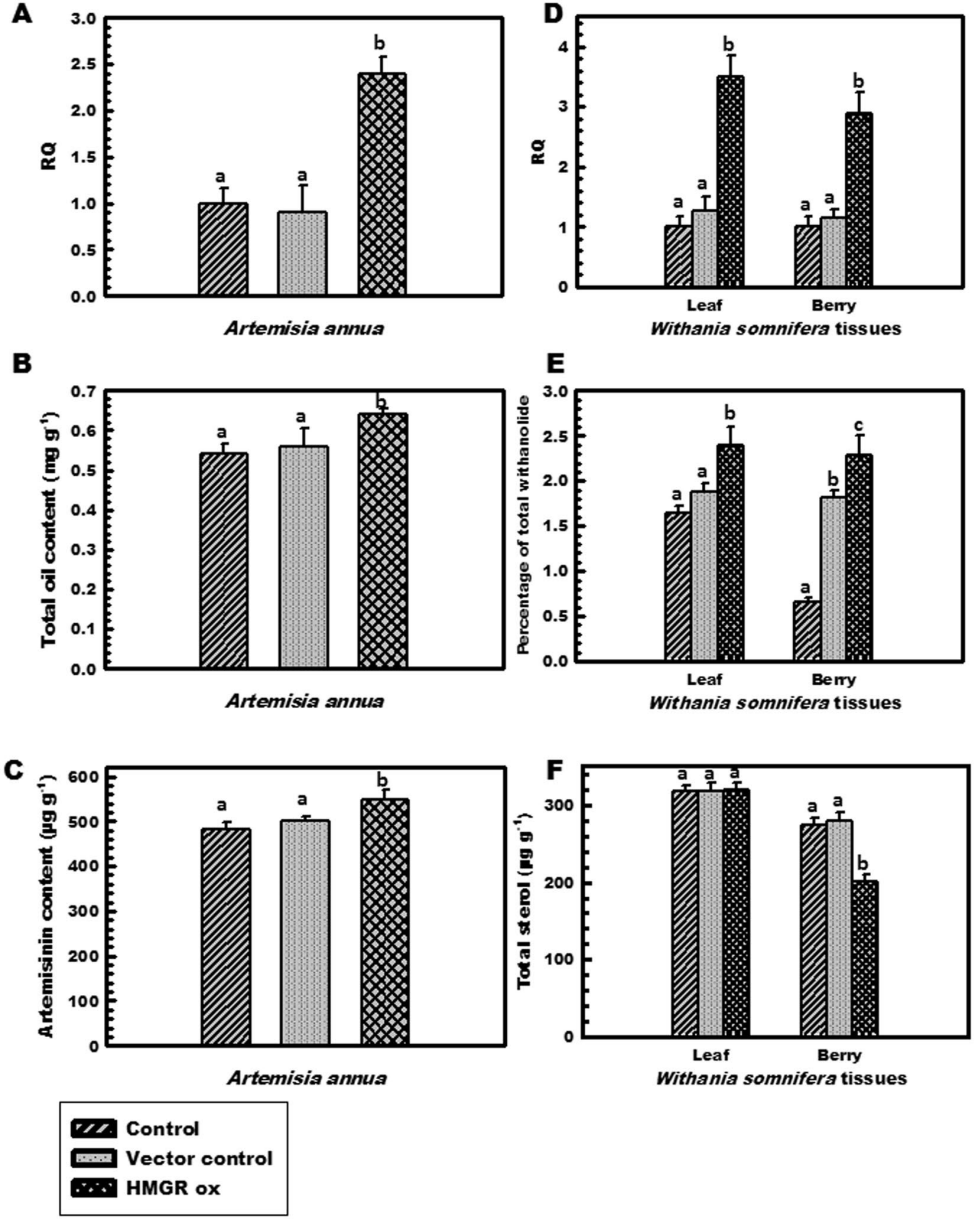
Expression profiles of *OkHMGR* transcript and secondary metabolite content in control and over-expressed lines of *A*. *annua* (**A**) *OkHMGR* mRNA transcript levels, (**B**) total essential oil content (mg/g FW g^−1^), (**C**) artemisinin content (µg g^−1^); and *W*. *somnifera* (**D**) mRNA transcript levels in leaf and berry, (**E**) total withanolide content (%), (**F**) total sterol content in leaf and berries (µg g^−1^). Control represents the untransformed tissues, vector control represents the tissues transformed with pCAMBIA1303 vector and *HMGR* ox represents tissues transformed with *OkHMGR*.

*A. annua* was selected as it contains important sesquiterpenoidal artemisinin as well as essential oil. Expression study revealed an increase of 2.4 folds in transformed tissues, than both untransformed control and vector control (Fig. 6A). In correlation to RT-PCR, total essential oil content was also high with a concentration of 0.64 mg/g which is 0.10 mg/g more than that of untransformed control and 0.08 mg/g more than vector control (Fig. 6B). HPLC analysis of untransformed control and over-expressed tissues showed an increase in artemisinin content by 7.4% (Fig. 6C).

In order to unravel the role of HMGR in synthesis of more complex higher phytomolecules of triterpenoid ancestry, *W*. *somnifera* was selected being rich in withanolides and sterols. Consistent with the results noted in *O*. *kilimandscharicum* and *A*. *annua*, the expression levels of *HMGR* was higher in over-expressed *W*. *somnifera* tissues than control tissues by 3.5 and 2.8 folds in leaves and berry respectively (Fig. 6D). To understand the linkage between *HMGR* expression and secondary metabolite biosynthesis, withanolides and sterols were extracted from the over-expressed as well as control tissues of the plant. Over-expression of *HMGR* is likely to drive the MVA isoprenoid biosynthesis in withanolides and sterols pathway; however we observed that the responses were tissue dependent. In both the tissues, over-expression led to increase in total withanolide content by upto 44% with a minor increase in total sterol content i.e. 0.5% (Fig. 6E, F). Transformation with vector control did not produce significant alterations in gene expression levels and withanolide and sterol content.

### Total carotenoid content in Ocimum species, *A. annua* and *W. somnifera*

To determine the effect of *HMGR* on carotenoids, the leaves of the plants were transiently transformed with *OkHMGR*. It was observed that similar to other metabolites of terpenoid pathway, carotenoids contents were increased after over-expression suggesting role of HMGR. The increase in carotenoid content was in the range of 3–11% in different *Ocimum* species. Increase in carotenoids was more in other plants in comparison to *Ocimum* species such as *A*. *annua* where upregulation was highest among all the plants studied, followed by *W*. *somnifera* leaf, with 33% increment. This may be because in *Ocimum* species major flux is diverted for the synthesis of monoterpenes and sesquiterpenes rather than carotenoids (Supplementary Fig. 6).

### Effect of inhibitors on gene expression and secondary metabolite content

**Figure 7:**
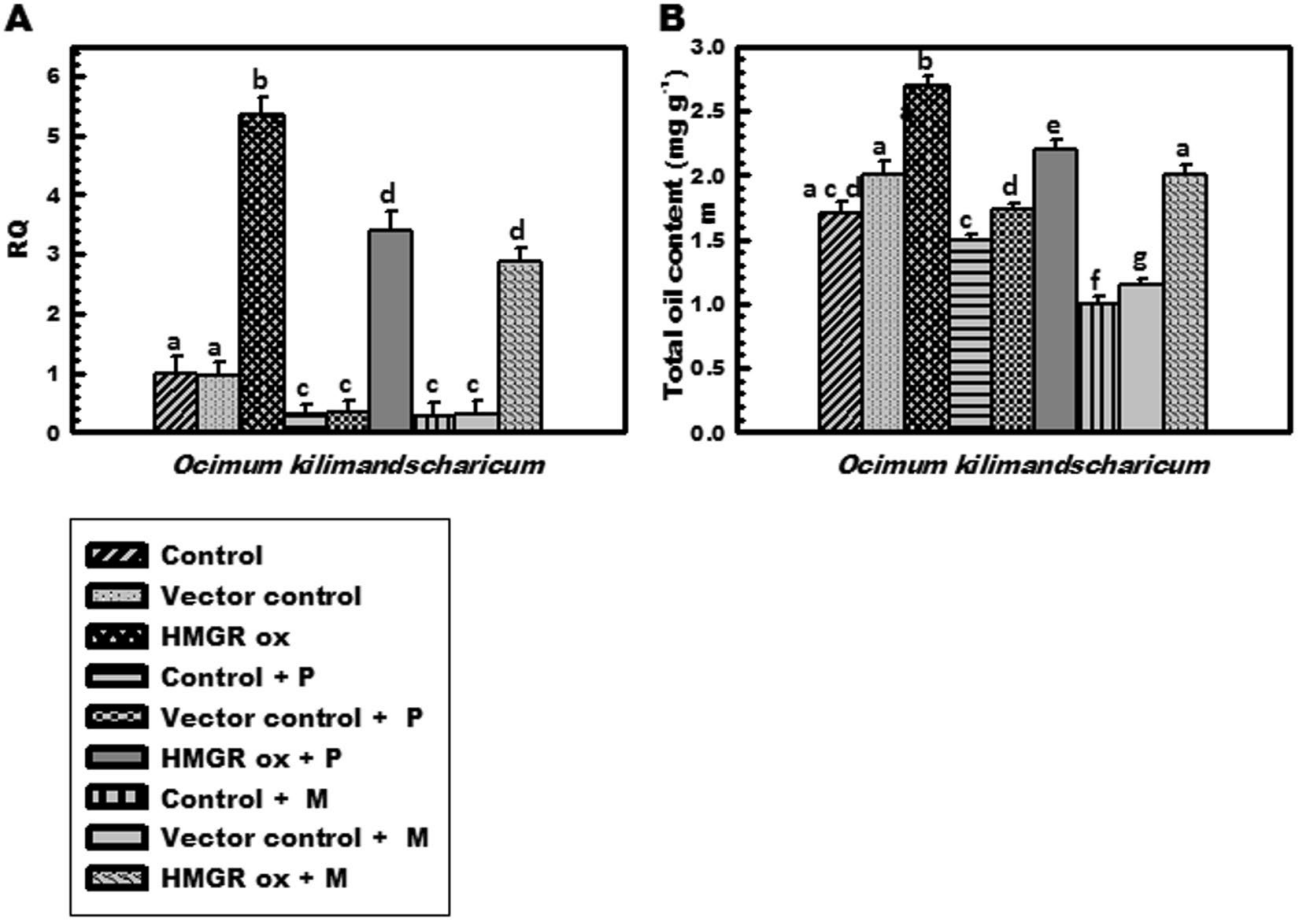
Analysis of secondary metabolites content and *HMGR* transcript expression levels in transiently transformed tissues of *O*. *kilimandscharicum* in presence and absence of inhibitors. (**A**) mRNA transcript levels and (**B**) Total essential oil content (μg g^−1^) in *O*. *kilimandscharicum*. Control- untransformed tissue without inhibitor, Vector control- pCAMBIA1303 vector transformed tissue without inhibitor, *HMGR* ox- transformed tissue without inhibitor treatment, Control + P- untransformed tissue with pravastatin treatment, Vector control + P- vector transformed tissue with pravastatin treatment, *HMGR* ox + P- transformed tissue with pravastatin treatment, Control + M- untransformed tissue with mevastatin treatment, Vector control + M- vector transformed tissue with mevastatin treatment and *HMGR* ox + M- transformed tissue with mevastatin treatment.

Inhibitors mevastatin and pravastatin were used to evaluate the blocking of the pathway and its impact on transformed and untransformed tissues. In *O*. *kilimandscharicum*, addition of statins resulted in down regulation of gene expression levels and total essential oil content in comparison to control untreated tissues (Fig. 7A,B). Treatment of over-expressed lines with these inhibitors showed dual action in the way that though addition of statins lowered the gene expression from 5.3 folds to 2.8–3.3 folds and total essential oil content by 18–25% in comparison to untreated over-expressed lines, but they still retained high expression to an extent, as the transcript abundance and total essential oil content was higher in over-expressed tissues than that of treated control (Fig. 7A,B).

**Figure 8:**
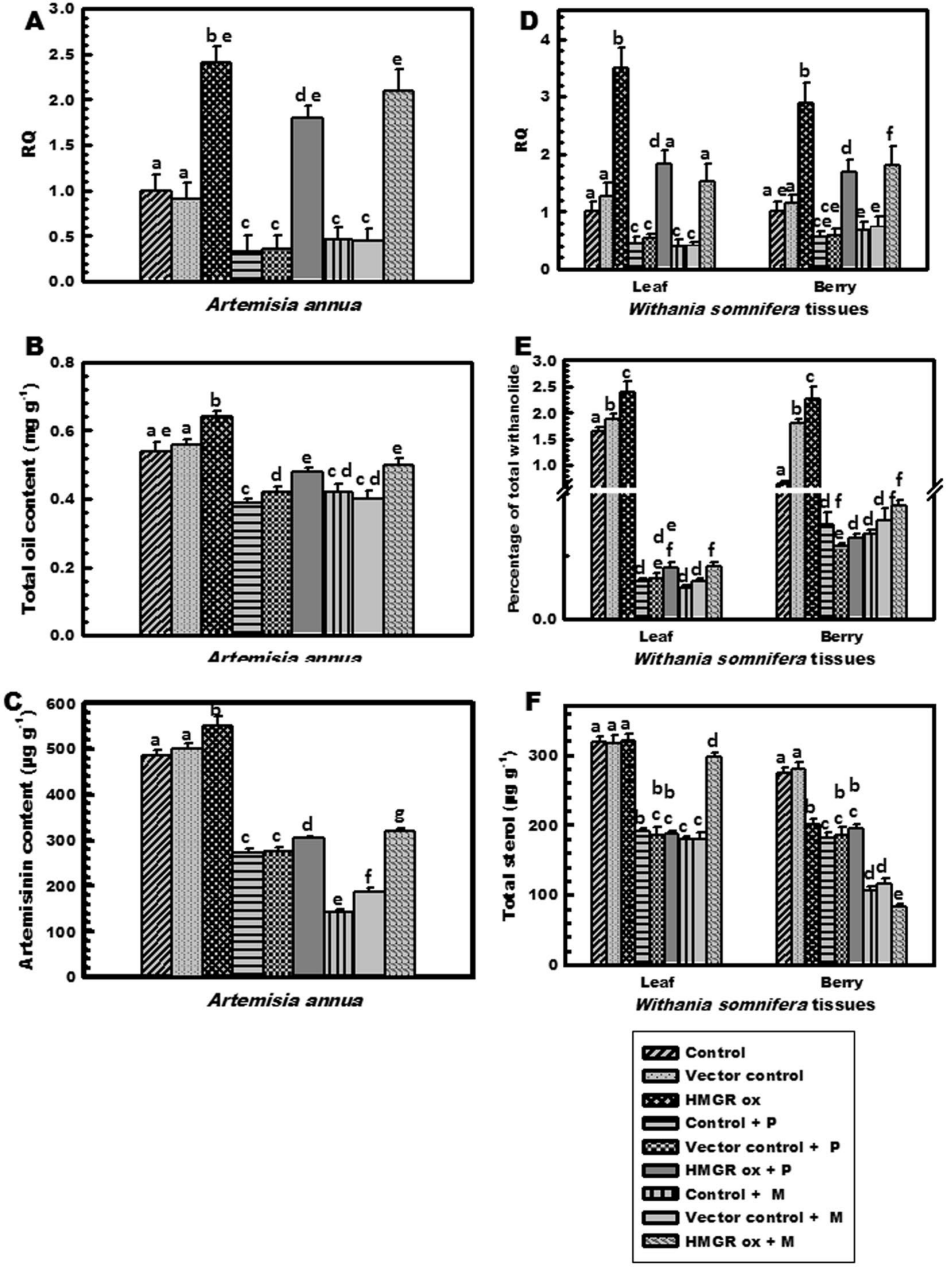
*OkHMGR* transcript expression and secondary metabolite analysis in transiently transformed tissues of *A*. *annua* and *W*. *somnifera* with and without inhibitors. (**A**) *HMGR* transcript levels, (**B**) Total essential oil content (mg g^−1^) and (**C**) Artemisinin content (µg g^−1^) in *A*. *annua*; (**D**) *HMGR* transcripts in tissues of *W*. *somnifera*, (**E**) total withanolide content (%) and (**F**) total sterol content (µg g^−1^). Control- untransformed tissue without inhibitor, Vector control- pCAMBIA1303 vector transformed tissue without inhibitor treament, *HMGR* ox- transformed tissue without inhibitor treatment, Control + P- untransformed tissue with pravastatin treatment, Vector control + P- vector transformed tissue with pravastatin treatment, *HMGR* ox + P- transformed tissue with pravastatin treatment, Control + M- untransformed tissue with mevastatin treatment, Vector control + M- vector transformed tissue with mevastatin treatment and *HMGR* ox + M- transformed tissue with mevastatin treatment.

Similar pattern was observed in *A*. *annua*, where inhibitor treatment lowered the relative expression to 0.3 and 0.4 fold for pravastatin and mevastatin treated and control lines, respectively (Fig. 8A) and a reduction in total essential oil content and artemisinin was also observed (Fig. 8B,C). Comparative analysis of treated transformed tissues with transformed control tissues, showed a decrease in mRNA transcript levels, total essential oil content as well as artemisinin. Compared to non-transgenic control, inhibitor treated over expressed lines retained their over-expression partly when gene transcript levels were determined which was found to be 1.8 folds higher than that of untransformed control. However, the secondary metabolites i.e. essential oil and artemisinin content declined, revealing the alterations in artemisinin were more prominent indicating preference of MVA pathway participation in biosynthesis (Fig. 8A,B,C).

*W*. *somnifera* was also influenced by the inhibitory action of inhibitors. Pravastatin and mevastatin treatment reduced the gene expression to 0.3–0.6 folds in untransformed treated control tissues. Overall decrease in both withanolides and sterol was observed in both tissues of *W*. *somnifera* after inhibitor treatment. In case of control leaves, there was prominent (about 96%) reduction in total withanolide content and 39–43% reduction in total sterol content compared to untreated untransformed tissues. Over-expressed lines treated with inhibitors showed lowering in gene expression levels, withanolide and sterol content to varying extents in comparison to untreated but transformed tissues (Fig. 8D,E,F).

## Discussion

Plants are rich source of secondary metabolites exhibiting important medicinal properties. Secondary metabolites of terpenoid origin are synthesized by mevalonate and non-mevalonate pathways and many studies have shown their involvement in the biosynthetic pathway^17–20^. Present study focusses on conversion of 3-hydroxy-3 methyl glutaryl Co-A into mevalonic acid by *OkHMGR* into an array of terpenoidal secondary metabolites such as monoterpenes, sesquiterpenes, triterpenoids and other isoprenoid compounds. These secondary metabolites are distinct in terms of number of carbon atoms, structure, functions, their targets and mode of action^21^. *HMGR* has been cloned and characterized in a number of plants such as *W*. *somnifera*^12^, *A*. *thaliana*^17^, *Andrographis paniculata*^19^ etc, however no reports are available for its cloning, kinetic and functional characterization in any of the *Ocimum* species. Realizing its importance and lack of knowledge about its role in *Ocimum* species, we isolated *HMGR* from *O*. *kilimandscharicum*, the Camphor Tulsi (the least explored species of *Ocimum*). Among various species, we primarily focused on *O*. *kilimandscharicum* as it contains more terpenoids than any other *Ocimum* species, which are known for the presence of phenylpropanoids. The isolated OkHMGR with a molecular mass of 60 kDa (Supplementary Table 1) showed evolutionarily relatedness to *S*. *miltiorrhiza* with highest similarity. The characterstic motifs such as HMG-CoA binding motif (TTEGCLVA and EMPVGYVQIP) and NADPH binding motifs (DAMGMNM and GTVGGGT) in OkHMGR sequence revealed that OkHMGR shares the conserved motifs with other plant species (Fig. 2A).

HMGRs have been broadly classified under two categories: class I and class II. Class I are found in eukaryotes and, are membrane bound while class II in prokaryotes and are soluble in nature^22^. OkHMGR consisted of two transmembrane domains at the N-terminal region and these transmembrane regions are believed to be responsible for anchoring the protein to the endoplasmic reticulum^23^. The N terminal region of OkHMGR is highly diverse while C terminal is conserved; these findings are in accordance to the other plant species *viz*. *Corylus avellana*, *Centella asiatica*^24,25^. HMGR protein exists as a tetramer where four monomers are arranged into two set of dimers, each dimer contains two active sites formed by residues from both monomers. Each monomer is further divided into three domains: N, L and S-domain. N domain is small consisting of amino helix and L-domain is unique for binding of HMG CoA. This domain is central and large consisting of regular expression of (TTEGCLVA) and (EMPVGYVQIP) while the S-domain (DAMGMNM), forms the binding site for NADPH^9^.

Biochemical characterization of the HMGR through its catalytic properties provided a detailed account of the kinetic nature of the protein as revealed by K_m_, V_max_, optimum pH, optimum temperature etc. Variation in the catalytic activity due to change in pH is a consequence in pattern of ionic bonding between the substrate and enzyme under acidic or alkaline conditions (Supplementary Fig. 4). Deviation in pH from optimal value leads to breakage of these bond and hence loss of active site and subsequently loss in enzymatic activity. pH optima was close to effective pH for *A*. *thaliana* i. e. 7.5^17^, whereas *Trypanosoma cruzi*, a parasite had optimal pH in the range of 5.7–6.3^26^. Lower value of K_m_ of HMG-CoA (88 µM) than NADPH (903 µM) showed that the enzyme has more affinity for its substrate rather than its cofactor. Other reports on HMGR where the enzyme had higher affinity for HMG-CoA than NADPH such as in *A*. *thaliana*, *Raphanus raphanistrum*, *Solanum tuberosum* with lower K_m_ for HMG-CoA as compared to Km for co-factor NADPH^17^. This clearly suggests that the availability of the substrate could be a rate controlling factor.

In *O*. *kilimandscharicum*, HMGR exhibited differential pattern of gene expression in different tissues of the plant. Similarly, in other plants such as in *C*. *arachnoids*, highest expression in stem tissues and lowest in leaves was recorded^27^. In *S*. *miltiorrhiza*, *HMGR* expression was detected in roots, stems and leaves but highest expression was found in roots, followed by stem and leaf tissues^28^. In, this study, high expression levels in flower tissues indicate that *OkHMGR* may have role in growth and development of flower or metabolite accumulation in inflorescence^29^ (Fig. 4A) as *HMGR* is involved in the synthesis of diverse metabolites *viz* sesquiterpenes, triterpenes and polyterpenes in the cytoplasm^30^. This varied response of *HMGR* in different plant tissues may be interpreted as the need of plant adjustments for various tissue specific roles, developmental and environmental conditions.

MeJA and SA are the plant signaling molecules which apart from having major role in defense responses are also involved in seed germination, fruit ripening and senescence which is a part of plant growth and development responses^31,32^. These molecules induce the expression of genes involved in secondary metabolism, cell-wall formation, stress protection and defense related processes^32^. Upregulation of *OkHMGR* in the presence of these signaling molecules (Fig. 4B,C) indicats that *HMGR* has a modulating function in response to various biotic and abiotic stresses and also key participation in physiological activities. These results were in agreement with the study done in *W*. *somnifera* where *HMGR* was responsive to MeJA and SA treatments^12^. Similarly, in *A*. *paniculata*, exogenous application of JA increased HMGR activity and secondary metabolite andrographolide content was also enhanced in jasmonic acid treated tissues^19^. Unlike other reports on drastic increase in HMGR activity on wounding, no significant changes in *OkHMGR* levels were observed. Apart from these elicitors certain hormones are known to influence the activity of enzymes. Application of GA_3_ increased the expression of *OkHMGR* transcripts (Fig. 4E). These results were consistent with the findings in *Pisum sativum* seedlings and *Cannabis sativa* where GA_3_ increased HMGR activity^33^. Thus it indicates that *OkHMGR* has hormonal control as well in the regulation of isoprenoids.

Stress induces a number of physiological and metabolic changes in the plant at primary level such as production of reactive oxygen species, changes in structure of chromatin, altering membrane permeability, modulation at transcriptional level, and hormone release etc^34^. Nature has evolved mechanisms so as to sustain itself under various stress conditions. Increase in transcription levels and hence enhanced production of secondary metabolites is one such possibility. Phytomolecules are reported to minimize the detrimental effects of stress by increasing the tolerance of the plant against stress and reducing reactive oxygen species production. Elevated terpenoids accumulation and higher expression of *HMGR* in *A*. *annua* was reported under prolonged water deficit stress and NaCl salinity stress in relation to adaptive response^35,36^. Temperature related stress modulates the synthesis of secondary metabolites such as phenolics, terpenoids, flavonoids^37^. These isoprene units such as α-pinene, have been known to withstand the harmful effect of temperature related stress. Monoterpenes and sesquiterpenes content increased in *S*. *lycopersicum* when the plant underwent heat and cold stress thereby suggesting that plants exhibit a definite set of cellular and metabolic responses necessary for the survival of the plant under stress conditions^38,39^. *HMGR* is an important step which is rate limiting in the biosynthesis of secondary metabolites. Production of secondary metabolites, influential in combating stress, is governed by expression of *HMGR* of mevalonate pathway. Varying pattern of *HMGR* expression manifests that *HMGR* has a shielding effect on plants in response to various stress by synthesizing various isoprenoids including sterols under stress conditions.

Transient transformation of a gene in plants offers various advantages due to which it has become a method of choice for determination of function of a gene. This approach is specifically beneficial for recalcitrant plants for regeneration, as it facilitates assaying gene transfer independent of regeneration^40^. Transient transformation of amorpha-3, 11-diene synthase (ADS) and epicedrol synthase (ECS) of *A*. *annua* in *N*. *benthamiana*, intermediate enzymes of artemisinin biosynthesis pathway was reported to show remarkable increase in the artemisinin production^41^. The percentage of the essential oil constituents in over expressed tissues was higher^4,42^ along with 5 folds higher transcripts in over-expressed tissues. Another report from *Parthenium argentatum* (guayule) also showed that the over-expression of *HMGR* led to 65% increase in polyisoprenoids rubber^43^. Interestingly, when change in individual constituents was being studied we noted that not only sesquiterpenes like caryophyllene, germacrene D but also some of the monoterpenes such as beta-myrcene, limonene, beta ocimene, camphene also increased^44,30^. The stable transformed plants of *O*. *kilimandscharicum* confirm the above results (data not shown). The moderate enhancement in monoterpenoid may account probably to the observations that an immediate conversion of the C5 moieties might have been influenced, although major impact was shed on MVA dependent metabolites. To further affirm our findings, the gene was transformed in different species of *Ocimum* namely, *O*. *basilicum*, *O*. *sanctum* and *O*. *gratissimum*, which represent another class in terms of phytochemical moieties. In all the three species over-expression led to the increase in total essential oil content, individual essential oil constituents and mRNA transcript levels. It is reported earlier that the monoterpenoid essential oils specifically ocimene are derived from MEP pathway as confirmed by ^13^C labelling and inhibitor treatment studies^45^. Interestingly, it was reported that in ripen strawberry, both monoterpenoid (linalool) and sesquiterpenoid (nerolidol) are biosynthesized in the cytosol as the gene FaNES1 which is capable of synthesizing both (linalool) and (nerolidol) depending upon the presence of its precursors GPP and FPP lacks plastid targeting peptide sequence at N-terminal. The observations were further confirmed by feeding studies where labeling of mevalonic acid led to labeling of linalool^46^. It was shown that the two isoforms of *A*. *thaliana* GPP synthase are involved in organelle specific manner, one being targeted to the plastids while the other was functionally linked to cytosol^47^. Shikonin, a monoterpene derivative from *L*. *erythrorhizon* is also produced by MVA pathway and negatively regulated by mevalonin treatment^48^. The reason which can be anticipated for this is loss of plastid targeting peptide due to some splicing event. Another reason is presence of dual isoforms of a synthase targeted to different cellular compartments which arises due to some mutation or difference in selection of first or second methionine group for initiation of protein synthesis or other post translational modifications^49^. The importance of *HMGR* is not restricted to triterpenoids only but carotenoids were also affected by its expression. Over-expression of *HMGR* increased the total carotenoids by up to 43% (Supplementary Fig. 6). As with monoterpenes, carotenoids are also assumed to be solely derived by MEP pathway but change in carotenoids content with change in *HMGR* expression shows an association between *HMGR* and carotenoids. Differential increment among various plants can be attributed to the physiology of the plant which varies considerably. *HMGR* synthesizes a pool of IPP which is acted by a number of enzymes of downstream pathway leading to production of various metabolites. It depends on the flux of these downstream enzymes which determine the metabolite to be synthesized and their levels.

Results in *O*. *kilimandscharicum* and different *Ocimum* species suggested to explore the *HMGR* function in plants of other family. *A*. *annua* is rich in essential oils and artemisinin content and over-expression of *HMGR* increased the total essential oil content by 15% while artemisinin content by 7.4%. Our result was in consensus with what has been observed earlier where transformation of plant with *Catharanthus roseus* (L.) *HMGR* could drive the artemisinin content^50^. These findings further proved that *HMGR* is involved in synthesis of sesqui-terpenes, especially artemisinin. Similarly, *W*. *somnifera* contains withanolides and sterols as an important phytoconstituents and *HMGR* is known to be involved in the production of various triterpenoids and sterols. In *W*. *somnifera*, over-expression of *OkHMGR*, transiently led to increase in mRNA transcripts and hence withanolides and sterols as well.

The application of inhibitor strategy onto untransformed control, vector control and over-expressed lines exhibited decrease in *HMGR* transcripts as well as terpenoid production. In transformed lines, over-expression and inhibition both the phenomenon were acting together as inhibition in transformed lines was less than that of control lines. These results were common in all the plants studied thereby confirming that *HMGR* has a major function in the synthesis of terpenoids. However, in case of *W*. *somnifera*, inhibitor treatment was more pronounced than over-expression while in some change in withanolide content was more than that of sterol content. The variation may arise due to the fact that triterpenoids and sterols are not the immediate products of *HMGR*. They are intervened by a series of enzymes and their intermediates which are finally converted to product. The plant tissues are acted upon by different downstream enzymes which drive the metabolite pool differentially towards the biosynthesis of terpenoids depending upon the conditions such as over-expression, silencing, inhibitor treatments etc. It has already been established that different tissues and chemotypes of *W*. *somnifera* have different withanolide content which to a great extent is governed by *HMGR* thereby imparting its role in withanogenesis^12,51^. Increased *HMGR* expression with diversion in pathway it selects i.e. either withanolides or sterols may also be due to post-transcriptional modifications of *HMGR*. Involvement of *HMGR* in triterpenoid synthesis was earlier reported when over-expression of *P*. *ginseng HMGR* in *A*. *thaliana* increased the content of sterols and triterpenoids, and in *P*. *ginseng* increased ginsenosides^52^. Although MVA dependent pathway has major participation in sterol and triterpenoid biosynthesis^51,52^, yet MEP pathway too has a role with extent, depending on the class of terpenoids^51^. Our earlier report on *W*. *somnifera* has established that major contribution was from MVA pathway rather than MEP pathway^25^.

In conclusion, a novel regulatory *HMGR* from less explored *O*. *kilimandscharicum*, is reported which has active participation and regulatory role in MVA derived phytomolecules not only in native plant which has terpenoid rich essential oil but in other medicinally important plants such as *W*. *somnifera* and *A*. *annua*. This is the first report of transformation of a gene in *O*. *kilimandscharicum* and hence opens-up new avenues for determination of function of more pathway genes downstream *HMGR* thereby providing a better understanding of biosynthetic pathway. The study also provides a novel *OkHMGR* which can be used as a powerful sequence to drive MVA led isoprenoids in medicinally and aromatically important plants.

## Materials and Methods

### Isolation of putative *OkHMGR* from leaf tissue

Total RNA was extracted from *O*. *kilimandscharicum* plants growing at CSIR-Central Institute of Medicinal and Aromatic Plant, Lucknow^53^. RNA was analyzed quantitatively and qualitatively and used for cDNA preparation using RevertAid First Strand cDNA Synthesis Kit (Thermo Fisher Scientific, Waltham, MA) according to manufacturer’s protocol. Degenerate primer HMGRDGF and HMGRDGR were designed (Supplementary Table 2) and used to amplify the partial *HMGR* fragment. The conditions used for amplification were: initial denaturation at 94 °C for 2.30 min followed by 35 cycles of 94 °C for 30 s, 54 °C for 40 s and 72 °C for 2 min followed by final extension for 7 min at 72°C in a thermal cycler (Eppendorf). Partial *OkHMGR* sequence was used to design 3′ and 5′ RACE primers. To amplify putative downstream region, PCR reaction was performed with 3′ RACE primer pair of *OkHMGR*DF1 and oligodT (for primary reaction) and *OkHMGR*DF2 and oligodT (for nested PCR reaction) under similar conditions as used for partial fragment with change in annealing temperature i.e. 52 °C. For amplification of upstream region, 5′ RACE primer pairs *OkHMGR*UR1 and AAP (for primary reaction) and *OkHMGR*UR2 and AUAP (for nested PCR reaction) were used with difference in annealing conditions i.e. 58 °C for 45 s. 50 µl reaction volume was set up for PCR reactions containing 100 ng template, 20 picomole of primers, 25 µl master mix and remaining volume was maintained using nuclease free water. Amplified fragments of both upstream and downstream regions were analyzed, eluted from gel, cloned in pJET1.2 cloning vector and transformed in DH5α *E*. *coli* cells. Transformed colonies were screened *via* colony PCR, plasmids were sequenced and confirmed as part of HMGR by BLAST analysis.

All the three sequences were assembled to make full length sequence of *OkHMGR*. Using this assembled full length sequence forward *OkHMGR*FLF and reverse *OkHMGR*FLR primers were designed containing *Sal*I and *Hind*III restrictions sites respectively for full length amplification of *OkHMGR*. The PCR conditions were as follows: 94 °C for 3.0 min, 35 cycles of 94 °C for 30 s, 55 °C for 1 min and 72 °C for 2.0 min followed by a final extension of 72 °C for 7 min. The resultant product was cloned in pJET1.2 cloning vector and transformed into DH5α competent cells. By colony PCR the positive clones of *OkHMGR* cDNA were screened. Full length *OkHMGR* was further confirmed by restriction digestion with *Sal*I and *Hind*III restriction enzymes.

### Sequence retrieval, multiple sequence alignment and phylogenetic studies

A total of 108 HMGR sequences from 55 plants along with *OkHMGR* were taken and multiple sequence alignment was done by the Mega 6.06^54^. Motifs and domains were assigned by the online web databases as MEME server^55^. Physiochemical properties were carried out, by using protpram tool from expasy server (https://web.expasy.org/protparam/). Secondary structure prediction was carried by using GORIV sever, widely used for modeling purposes (https://npsa-prabi.ibcp.fr/cgi-bin/npsa_automat.pl?page = /NPSA/npsa_gor4.html).

### Heterologous expression of *OkHMGR* in *E*. *coli* and purification of recombinant OkHMGR protein

Plasmid DNA of pJET cloned full length *OkHMGR* and pET28a plasmid were digested by *Sal*I and *Hind*III restriction enzymes and both were ligated by T4 DNA ligase. Ligated product, pET-*OkHMGR*, was transformed in BL21 (DE3) competent cells and confirmed by colony PCR and restriction digestion. For over-expression of pET-*OkHMGR* in *E*. *coli* (BL21) the cells were induced by 0.8 mM isopropyl β-D-1-thiogalactopyranoside (IPTG) and allowed to grow at 18 °C for overnight. The cell lysate was prepared by sonication and analysed by12% SDS gel. For purification of recombinant OkHMGR enzyme, one liter recombinant *E*. *coli* BL21 (DE3) culture was induced and purified through Ni-NTA affinity column chromatography. Fractions obtained after elution were screened for protein concentration (A_280_) as well as for its catalytic activity.

### Enzyme assay

HMG-CoA-dependent oxidation of NADPH *via* HMGR enzyme was carried out at 30 °C using spectrophotometer at 340 nm essentially by using the method of Dale *et al*.^17^. Assay mixture consisted of 0.05 mM HMG-CoA, 3.0 mM NADPH, 7.5 mM dithiothreitol (DTT) and 150 mM potassium phosphate buffer (pH 7.0). Absorption coefficient of 6220 M^−1^ cm^−1^ at 340 nm for NADPH was used for computation of activity. Further, various catalytic properties of OkHMGR enzyme were estimated by altering required parameters.

### Expression profiling of *OkHMGR*

Relative level of *OkHMGR* transcripts was determined in six different tissues of the plant i.e. young leaf, middle/mature leaf, large/fully mature leaf, stem, bud and flower as described earlier^56^. Gene specific qRT (quantitative real time) primers were designed using Beacon Designer Software (Supplementary Table 2). PCR reaction mixture comprised of ~50 ng cDNA, 5 pmole of each forward and reverse primers, 5 µl of Power SYBR Green PCR master mix 2X (Applied Biosystems, USA) and the final volume was maintained with nuclease free water to 10 µl. Actin gene was taken as an endogenous control^52^. All the reactions were carried out in triplicates. The reactions were carried out on StepOne^TM^ Real Time PCR system with 48-well block module. The relative gene expression levels were calculated by ΔΔCT method and expressed as relative quantification (RQ) values. For different stresses and elicitor treatments leaves were treated for 4 hours. In case of wounding the leaves were injured with scalpel blade at different sites and treated samples were then collected after 0.5 h, 3 h and 24 h. Heat and cold stress were given at 55 °C and 4 °C respectively for different time durations i.e. 0.5, 1, 2, 4 hrs. Leaves were also treated with NaCl (0, 50, 100 and 250 mM), salicylic acid and methyl jasmonate (0, 10, 50, 100 and 500 µM), and gibberellic acid (0, 1, 10 and 50 µM) for 3 hrs. All the tissues were subjected to RNA isolation, cDNA preparation and transcript profiling.

### Over-expression of *OkHMGR*

For transient expression of *OkHMGR* in plants, the coding sequence of gene was cloned in plant binary vector pCAMBIA1303. Coding sequence of *OkHMGR* cloned in pJET 1.2 cloning vector, was digested with *Bgl*II enzyme. The resulting fragment was cloned at *Bgl*II site of pCAMBIA1303 under the control of CaMV35S promoter. After confirmation of ligation event through colony PCR and digestion, the resulting pCAMBIA:*OkHMGR* construct was transformed in *A*. *tumefaciens* strain GV3010 *via* freeze thaw method^57^. Transformation of pCAMBIA:*OkHMGR* construct in *A*. *tumefaciens* was confirmed by colony PCR with *npt*II primers (Supplementary Table 2). To check the activity of *OkHMGR* at transient level, *O*. *basilicum*, *O*. *sanctum*, *O*. *gratissimum*, *O*. *kilimandscharicum*, *W*. *somnifera* and *A*. *annua* were used. The leaves from these plants were processed for transformation as reported earlier^18^. For inhibitor studies, tissues of control and transiently transformed plants were treated with both mevastatin and pravastatin individually for 24 hrs followed by various analysis.

### Secondary metabolite determination

Essential oil from control as well as transformed tissues was isolated by the method described in Bose *et al*.^58^. Essential oil constituents were analyzed by using GC and GC-MS analysis as reported earlier for *A*. *annua*^35,36^. Artemisinin content was determined by the protocol as mentioned in our previous publications^27^. Withanolide content was determined by our earlier protocol established for *W*. *somnifera*^59,60^.

### Experimental design and statistical analysis

Each experiment was set in triplicates. To test statistical significance of difference among mean responses at a significance of P = 0.05 one way Analysis Of Variance (ANOVA) was used.

## Electronic supplementary material

Supplementry Figures

Supplementary Tables
