# Supplementary material for: HMG-CoA reductase from Camphor Tulsi (Ocimum kilimandscharicum) regulated MVA dependent biosynthesis of diverse terpenoids in homologous and heterologous plant systems
Source: Sci Rep. 2018 Feb 23;8:3547. doi: 10.1038/s41598-017-17153-z (PMC5824918; doi:10.1038/s41598-017-17153-z)
Supplement: Supplementary file 1 — Supplementry Figures [file 41598_2017_17153_MOESM1_ESM.docx]

####

**HMG-CoA reductase from Camphor Tulsi (*Ocimum kilimandscharicum*) regulated MVA dependent biosynthesis of diverse terpenoids in homologous and heterologous plant systems**

**Shilpi Bansal^1, 2^, Lokesh Kumar Narnoliya^1^, Bhawana Mishra^1, 2^, Muktesh Chandra^1^, Ritesh Kumar Yadav^1^, Neelam Singh Sangwan ^1 *^**

***Correspondence to nsangwan5@gmail.com**

**Affiliation of authors**

1. Department of Metabolic and Structural Biology, CSIR-Central Institute of Medicinal and Aromatic Plants, Lucknow-226015, UP, India
2. Academy of Scientific and Innovative Research (AcSIR), New Delhi

**C**

**B**

**A**

**Supplementary Figure 1.** PCR analysis of *OkHMGR* (**a**) Partial fragments (500 bp) obtained from degenerate primers, (**b**) 5’ RACE amplification of *OkHMGR* (700 bp) and (**c**) 3’ RACE amplification of *OkHMGR* (1.0 kp). M: 100 bp ladder.


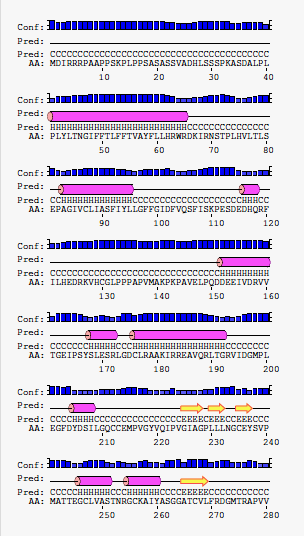

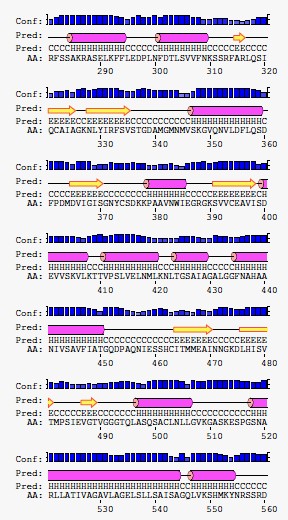

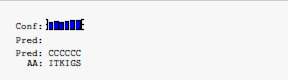

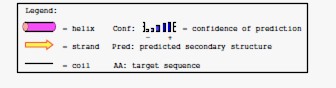


**Supplementary Figure 2.** Secondary structure of *Ocimum kilimandscharicum* HMGR enzyme.

**Supplementary Figure 3.** Effect of buffer on catalytic activity of HMGR of *O. kilimandscharicum.*

**Supplementary Figure 4.** Effect of pH on catalytic activity of HMGR of *O. kilimandscharicum.*

**Supplementary Figure 5.** Effect of ions on catalytic activity of HMGR of *O. kilimandscharicum.*

**Supplementary Figure 6.** Comparative profiling of total carotenoids in untransformed, vector transformed and *OkHMGR* transformed leaves of different plants *O. sanctum* (OS), *O. gratissimum* (OG), *O. basilicum* (OB), *O. kilimandscharicum* (OK), *A. annua* (AA), *W. somnifera* berry (WSB) and leaf (WSL).

|  | **M Ok** |  |
| --- | --- | --- |
|  |  |  |
|  |  |  |


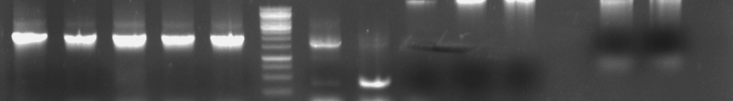
**Supplementary Figure 7.** PCR amplification of *OkHMGR* full length gene. Lane M and lane Ok of this figure has been reproduced in main Figure 3A. Other lane of this gel picture are extra and need not to be considered as a part of current study.

Figure in main manuscript

Original gel picture for Figure 3A

**Supplementary Figure 8:** Processed and unprocessed Figure 3A

Figure in main manuscript

Original gel picture for Figure 3B

**Supplementary Figure 9:** Processed and unprocessed Figure 3B
