# Supplementary material for: HMG-CoA reductase from Camphor Tulsi (Ocimum kilimandscharicum) regulated MVA dependent biosynthesis of diverse terpenoids in homologous and heterologous plant systems
Source: Sci Rep. 2018 Feb 23;8:3547. doi: 10.1038/s41598-017-17153-z (PMC5824918; doi:10.1038/s41598-017-17153-z)
Supplement: Supplementary file 2 — Supplementary Tables [file 41598_2017_17153_MOESM2_ESM.pdf]

HMG-CoA reductase from Camphor Tulsi (*Ocimum kilimandscharicum*) regulated MVA dependent biosynthesis of diverse terpenoids in homologous and heterologous plant systems

Shilpi Bansal[1, 2], Lokesh Kumar Narnoliya[1], Bhawana Mishra[1, 2], Muktesh Chandra[1], Ritesh Kumar Yadav[1], Neelam Singh Sangwan[1 *]

***Correspondence to nsangwan5@gmail.com, ns.sangwan@cimap.res.in**

Affiliation of authors

1. Department of Metabolic and Structural Biology, CSIR-Central Institute of Medicinal and Aromatic Plants, Lucknow-226015, UP, India
2. Academy of Scientific and Industrial Research (AcSIR), New Delhi

Supplementary Table1. Physiochemical properties of *OkHMGR*

**S.no**	**Parameter**	**Score**
1	MW	60534.8 Da
2	Theoretical pI	7.10
3	Negatively charged residues (Asp+Glu)	53
4	Positively charged residues (Arg+Lys)	53
5	Instability Index	43.45 (unstable protein)
6	Aliphatic index	95.46
7	Grand average of hydropathicity (GRAVY)	0.129
8.	GC content	G(25%; 526), C(24%; 503)

Supplementary Table 2. List of primers used for cloning and characterization of *OkHMGR*

Primer name	Sequence
HMGRDGF	5′TRGGRCARTGCTGYGARATGCC 3′
HMGRDGR	5′ACWGAYTTDCCWCGYCCTTCAATCCA 3′
OBHMGRUR1	5′CCTTCGATCCAGTTGACGG 3′
OkHMGRUR2	5′GAGCAYTAYTTMCCYGAYATGC 3′
OkHMGRDF1	5′MGCTCTYGGYGGMTTCAACG 3′
OkHMGRDF2	5′GARAGCTCTCACTGCATYACS 3′
OkHMFLF1	5′GTCGACATGGATATCCGCCGGAGGCC 3′
OkHMFLR1	5′AAGCTTTTAGGACCCAATCTTCGTGATGTCC 3′
OkHMGRRTF1	5′AGCCATAGAAGCTGGGAAGGACA 3′
OkHMGRRTR1	5′GCCAGAGCCTCTTTGAGCTCA 3′
*npt*II F	5′CTGAATGAACTGCAGGACGAGG 3′
*npt*IIR	5′GCCAACGCTATGTCCTGATAGC 3′
Actin FP	5′CTTTCTACAATGAGCTTCGTG 3′
Actin RP	5′ATACAGTGAGAGAGGACAGCCTG 3′
